# Preemptive intravenous iron therapy versus autologous whole blood therapy for early postoperative hemoglobin level in patients undergoing bimaxillary orthognathic surgery: a prospective randomized noninferiority trial

**DOI:** 10.1186/s12903-020-01359-1

**Published:** 2021-01-07

**Authors:** Min Suk Chae, Mihyun Lee, Min Ho Choi, Je Uk Park, Misun Park, Young Hoon Kim, Hoon Choi, Jin Joo, Young Eun Moon

**Affiliations:** 1 Department of Anesthesiology and Pain Medicine, Seoul St. Mary’s Hospital, College of Medicine, The Catholic University of Korea, 222, Banpo-daero, Seocho-gu, Seoul, 06591 Republic of Korea; 2 Department of Oral and Maxillofacial Surgery, Seoul St. Mary’s Hospital, College of Medicine, The Catholic University of Korea, Seoul, Republic of Korea; 3 Department of Biostatistics, Clinical Research Coordinating Center, Catholic Medical Center, The Catholic University of Korea, Seoul, Republic of Korea

**Keywords:** Anemia, Iron, Ferric carboxymaltose, Autologous whole blood, Bimaxillary orthognathic surgery

## Abstract

**Background:**

Previous studies have reported the efficacy and safety of intravenous (IV) iron therapy during the perioperative period as an alternative and adjunct to allogeneic blood transfusion. Preemptive IV iron therapy provides noninferior hemoglobin levels on postoperative day (POD) 1 compared to autologous whole blood therapy (AWBT) in healthy patients who had undergone bimaxillary orthognathic surgery.

**Methods:**

This was a prospective, patient-randomized, noninferiority trial. After excluding 2 patients, 64 patients were divided into two groups: the IV iron therapy group (patients received IV iron infusion 4 weeks before surgery; *n* = 32) and the AWBT group (2 units of autologous whole blood were collected 4 and 2 weeks before surgery; *n* = 32). The primary outcome was hemoglobin level on POD 1 and the prespecified noninferiority limit was − 1 g/dL.

**Results:**

Baseline data were comparable, including hemoglobin and iron levels, between the two groups. Immediately before surgery, the levels of hemoglobin, iron, and ferritin were higher in the IV iron group than in the AWBT group. The mean treatment difference (iron group—whole blood group) in hemoglobin level on POD 1 between the two groups was 0.09 (95% CI = − 0.83 to 1.0). As the lower limit of the 95% CI (− 0.83) was higher than the prespecified noninferiority margin (δ = − 1), noninferiority was established. On POD 2, the hemoglobin level became lower in the iron group, which eventually led to greater requirement of allogeneic blood transfusion compared to the whole blood group. However, the iron group did not require allogeneic blood transfusion during or early after surgery, and the whole blood group showed continuously higher incidence of overt iron deficiency compared to the iron group.

**Conclusion:**

As collection of autologous whole blood caused overt iron loss and anemia before surgery and intraoperative transfusion of whole blood was not able to prevent the occurrence of persistent iron deficiency after surgery, IV iron therapy was found to have potential benefits for iron homeostasis and subsequent erythropoiesis in healthy patients early after bimaxillary orthognathic surgery.

*Trial registration*: Clinical Research Information Service, Republic of Korea, approval number: KCT0003680 on March 27, 2019. https://cris.nih.go.kr/cris/search/search_result_st01_kren.jsp?seq=15769&sLeft=2&ltype=my&rtype=my.

## Background

Bimaxillary orthognathic surgery has been widely performed to correct bimaxillary prognathism or to reposition a mid-facial depression that is frequently associated with a higher risk for large-scale hemorrhage or transfusion requirement during surgery, because of the facial anatomy and surgical approach [1]. Strategies to decrease the use of allogeneic blood transfusion include preoperative autologous blood donation and intraoperative hypotensive anesthesia. Autologous whole blood therapy (AWBT) has been encouraged in healthy and young patients because of concerns about allogeneic blood transfusion-related complications, such as infection, allergic or anaphylactic reactions, and alloimmunization [2–4]. In surgeries where a large amount of blood loss is anticipated, such as cardiac or orthopedic surgery, intraoperative transfusion of autologous whole blood favorably enhances postoperative recovery after surgery, including reduced rates of respiratory failure, pneumonia, chest tube output, reoperation for bleeding or infection, and length of hospital stay [5, 6].

However, in whole blood donation studies [7, 8], donors have been reported to have lost large amounts of iron on harvesting of a unit of whole blood (500 mL, plus 25 mL for testing), and it requires more than 6 months to restore the iron to the level before donation with a standard diet. After regular blood donation, approximately 25–35% of blood donors become iron deficient, subsequently leading to side effects that can impact health, such as overt anemia, fatigue, neurocognitive changes, pica, and restless legs syndrome [6, 8–11]. Low-dose oral iron intake was reported to contribute to marked reduction of hemoglobin recovery time after donation compared to no oral iron intake, but full recovery of iron and hemoglobin levels took a long time [8]. A previous retrospective study suggested that a larger proportion of patients who received intravenous (IV) ferric carboxymaltose, as a dextran-free IV iron complex, experienced effective reversal of acute isovolemic anemia compared to those without treatment [12]. Compared to oral iron, high-dose IV iron infusion is also associated with faster and higher replenishment of depleted hemoglobin and iron levels without adverse events [13]. Many surgical studies have suggested that IV iron therapy before or after surgery may be efficacious and safe as an alternative and adjunct to allogeneic blood transfusion [10, 14–17]. However, no studies have confirmed this observation in patients who have undergone bimaxillary orthognathic surgery.

We hypothesized that IV iron therapy would provide noninferior hemoglobin levels on postoperative day (POD) 1 compared to AWBT, while reducing postoperative adverse effects of iron depletion and less discomfort/pain during treatment. Therefore, we conducted a randomized, noninferiority clinical trial on healthy patients who had undergone bimaxillary orthognathic surgery.

## Patients and methods

### Ethical considerations

**Fig. 1:**
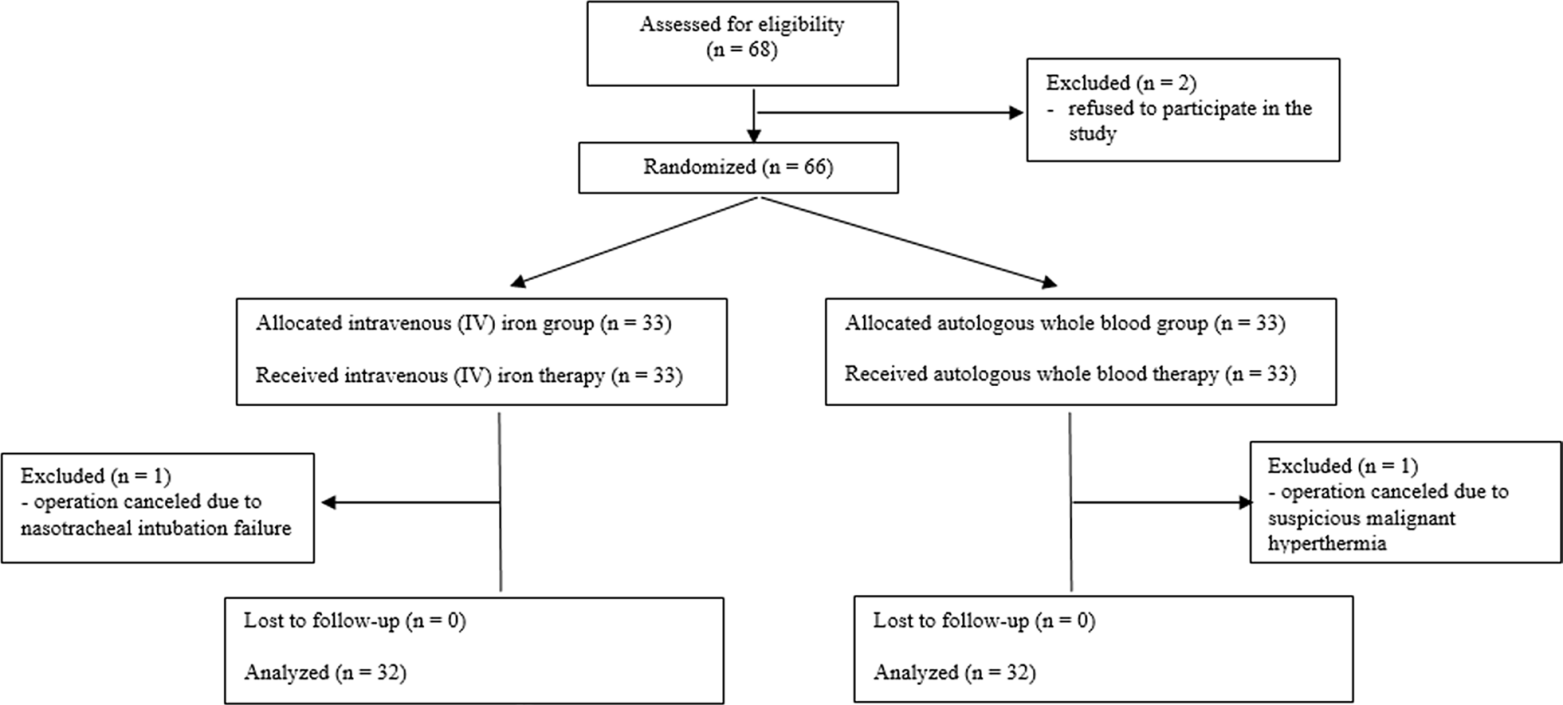
Consolidated standards of reporting trials (CONSORT) flow chart

This was a prospective, patient-randomized, noninferiority trial. The protocol was approved by the Institutional Review Board of Seoul St. Mary’s Hospital Ethics Committee (approval no. KC18MESI0583) on December 19, 2018. The study was performed in accordance with the principles of the Declaration of Helsinki. The study protocol was prospectively registered at a publicly accessible clinical registration site that is recognized by the International Committee of Medical Journal Editors (Clinical Research Information Service, Republic of Korea, approval number: KCT0003680) on March 27, 2019. Written informed consent was obtained from all patients at our hospital who were enrolled between March 2019 and May 2020. The study adhered to the Consolidated Standards of Reporting Trials (CONSORT) guidelines (Additional file 1) and a CONSORT flow chart is provided in Fig. 1. Additional file 2 presents a summary of our study protocol.

### Inclusion and exclusion criteria

The inclusion criteria for this study were as follows: age ≥ 20 years; scheduled for elective bimaxillary orthognathic surgery; and American Society of Anesthesiologists (ASA) physical status I or II [18]. Exclusion criteria were as follows: hemodynamic instability requiring rescue therapy, such as strong vasopressor infusion (i.e., epinephrine or norepinephrine); ASA physical status III–V; history of iron drug-related side effects, such as allergy; medication history with anticoagulants, such as aspirin, clopidogrel, or warfarin; anemia (i.e., hemoglobin level < 11 g/dL) [19, 20]; and refusal to participate in the study.

### Randomization

The patients were randomly divided into two groups: the IV iron group and the AWBT group. Randomization was performed using sealed, opaque envelopes. An independent colleague randomly grouped the envelopes in blocks of 10 with a 1:1 ratio to produce an equal distribution across the whole study period. The envelopes were stacked and stored. When an enrolled patient arrived in the treatment area, the upper envelope was opened by the attending physicians who performed the treatments, i.e., IV iron infusion or collection of whole blood. In the operating room, the attending anesthesiologist and nurses were aware of the group allocations, but they were not involved in future patient care or data collection.

### Treatment

In the iron group, IV 500 mg ferric carboxymaltose (Ferinject; Vifor Pharma, Glattbrugg, Switzerland) mixed with 100 mL normal saline was administered for 30 min 4 weeks before surgery in accordance with the manufacturer’s instructions.

In the AWBT group, two units of autologous blood were collected, 1 unit at a time with an interval of at least 2 weeks between collections; thus, each unit was collected on 4 and 2 weeks before surgery. The blood volume collected at one time was 320 mL, and therefore, the total amount of whole blood was 640 mL. It was stored in blood bags with a preservation solution of citrate–phosphate-dextrose-adenine at 4 °C until the day of surgery, which is a standard blood management of the hospital [19–22]. All collected whole blood was transfused intraoperatively using a warming infusion device.

During and after IV iron supplementation or AWBT, all patients were closely monitored for complications, such as fever (≥ 38 °C), phlebitis, nausea/vomiting, injection-site reactions (i.e., pain or urticaria), dizziness/syncope, hypotension (i.e., SBP < 90 mmHg), tachycardia (i.e., heart rate [HR] > 100 beats/min) or hypersensitivity/allergy.

### Surgery and anesthesia

Surgical technique and anesthetic care were as described previously [23]. Briefly, the care of patients was standardized between both groups apart from the treatments applied. Bimaxillary orthognathic surgery, including a bilateral sagittal split osteotomy and a Le Fort I osteotomy, was performed by an experienced surgeon, and balanced general anesthesia was provided without pre-medication by experienced attending anesthesiologists. To reduce surgical bleeding, induced hypotensive anesthesia with a systolic blood pressure (SBP) < 100 mmHg was achieved by intermittent IV boluses of sodium nitroprusside or nicardipine, especially between the beginning of osteotomy and the end of osteosynthesis. Hemodynamic monitoring, including SBP, diastolic blood pressure (DBP), HR, electrocardiogram, pulse oximetry, and end-tidal carbon dioxide pressure, were regularly recorded every 5 min during surgery. Blood lost by surgical hemorrhage was replaced to prevent hypovolemia with one bag (500 mL) of colloid product (6% hydroxyethyl starch, volulyte) in the IV iron group and by 2 units (640 mL) of autologous whole blood in the AWBT group.

Based on the practice guideline [19], allogeneic packed red blood cell (PRBC) transfusion was performed at the discretion of the attending anesthesiologists or physicians, when hemoglobin level reached between 6 and 10 g/dL with consideration of potential or actual ongoing hemorrhage, intravascular volume status, organ ischemic signs, or adequacy of cardiopulmonary reserve. These blood products were administered unit-by-unit.

### Clinical variables

Preoperative findings included demographic variables, ASA physical status, vital signs (i.e., SBP, DBP, and HR). Intraoperative findings included total operation duration, average vital signs during surgery, total amount of fluid input, including crystalloid and colloid, hemorrhage, and urine output. Laboratory variables (i.e., hemoglobin, hematocrit, red blood cell [RBC] count, mean corpuscular volume, mean corpuscular hemoglobin, mean corpuscular hemoglobin concentration, iron, ferritin, C-reactive protein, white blood cell count, neutrophil, lymphocyte, and platelet counts, international normalized ratio [INR], activated partial thrombin time, antithrombin III, and fibrinogen) were regularly measured four times: 4 weeks before surgery (baseline), immediately before surgery, and on PODs 1 and 2.

### Outcomes

The primary outcome was the hemoglobin level on POD 1. The secondary outcomes included the levels of inflammatory and coagulation variables on PODs 1 and 2, and changes in blood cell-related variables during POD 2. The incidences of anemia defined as hemoglobin level < 13 g/dL in males and < 12 g/dL in females, low iron level defined as < 50 μg/L, and low ferritin level defined as < 15 ng/mL in males and < 10 ng/mL in females were analyzed in the two groups [9, 19]. The degree of discomfort when iron was intravenously infused or whole blood was collected was analyzed using a visual analog scale (VAS; 0 = no discomfort and 10 = the worst discomfort). Surgical complications, such as allogeneic blood transfusion, were analyzed using Clavien–Dindo classification [24], and total hospital stay was measured in both groups.

### Statistical analyses

The sample size was calculated based on the primary endpoint according to the noninferiority hypothesis. The predetermined noninferiority limit (δ) was set to a difference in hemoglobin level of − 1 g/dL between the two groups (IV iron group—AWBT group) that was considered clinically acceptable by experts at our institution. Based on preliminary data, a standard deviation (SD) of 1.3 g/dL was assumed for the hemoglobin distribution. With α = 0.05 and power of 90%, 30 patients were required in each group. Assuming a 10% dropout rate, we decided to enroll 33 patients per group. For the primary outcome, the noninferiority of the IV iron therapy was considered if the lower boundary of the two-sided 95% confidence interval (CI) lay above the noninferiority margin of − 1 g/dL [25].

Values are expressed as the mean ± SD, median with interquartile range (IQR), or as numbers with percentages. The normality of the distribution of the continuous data was evaluated using the Shapiro–Wilk test. The perioperative findings were compared between the two groups using the unpaired *t* test or the Mann–Whitney *U* test, and Pearson’s *χ*^2^ test or Fisher’s exact test, as appropriate. Serial changes in RBC-related variables were analyzed using the paired *t* test or Wilcoxon’s signed rank test. All tests were two-sided, and *p* < 0.05 was taken to indicate statistical significance. Statistical analyses were performed using SPSS for Windows (ver. 24.0; IBM Corporation, Armonk, NY) and MedCalc for Windows software (ver. 11.0; MedCalc Software, Ostend, Belgium).

## Results

A total of 68 patients were assessed for eligibility and enrolled in this study. However, two patients refused to participate (*n* = 2). In addition, one patient with suspected malignant hyperthermia (*n* = 1), and one patient in whom nasotracheal intubation failed (*n* = 1), were excluded. Therefore, 64 patients were included in the analyses (Fig. 1). Our study population included healthy patients without comorbidities, such as hypertension, diabetes mellitus, and anemia. There were no adverse events related to IV iron therapy or AWBT, such as fever (≥ 38 °C), phlebitis, nausea/vomiting, injection-site reactions (i.e., pain or urticaria), dizziness/syncope, hypotension, tachycardia, or hypersensitivity/allergy, before surgery. There were no significant differences in preoperative and intraoperative findings between the two groups (Table 1).

**Table 1:** Comparisons of pre- and intraoperative findings between the two groups

Group	Intravenous iron	Autologous whole blood	*p*
n	32	32	
Preoperative findings			
Sex (male)	16 (50%)	17 (53%)	0.802
Age (years)	21 (20–25)	21 (20–24)	0.47
Height (cm)	170.0 (160.7–176.8)	169.5 (164.0–177.0)	0.527
Weight (kg)	60.5 (54.0–71.8)	63.0 (50.0–77.0)	0.591
Body mass index (kg/m^2^)	22.0 ± 3.9	22.6 ± 5.3	0.579
ASA physical status I	32 (100%)	32 (100%)	–
Vital signs			
Systolic blood pressure (mmHg)	116 ± 9	118 ± 13	0.325
Diastolic blood pressure (mmHg)	72 ± 10	71 ± 9	0.664
Heart rate (beats/min)	81 ± 12	81 ± 12	0.96
Intraoperative findings			
Total operation duration (min)	213 ± 39	230 ± 35	0.07
Average during surgery			
Systolic blood pressure (mmHg)	94 (90–97)	93 (88–99)	0.851
Diastolic blood pressure (mmHg)	52 (46–55)	53 (48–59)	0.371
Heart rate (beats/min)	79 (72–90)	78 (77–91)	0.51
*Total amount of fluid input (mL)*			
Crystalloid (mL)	1250 (1000–1975)	1500 (645–2250)	0.968
Colloid (mL)	650 (500–750)	–	–
Autologous whole blood (mL)	–	640 (640–640)	–
Hemorrhage (mL)	900 (500–1138)	1000 (700–1425)	0.424
Urine output (mL)	150 (120–250)	215 (50–400)	0.491

Values are expressed as mean ± SD, median (interquartile) and number (proportion)

*ASA* American Society of Anesthesiologist

### Comparison of perioperative red blood cell-related findings between the two groups

Four weeks before surgery (baseline) (Table 2), hemoglobin, hematocrit, RBC count, red cell index (i.e., MCV, MCH, MCHC), iron, and ferritin were comparable between the two groups. Immediately before surgery, the RBC count, hemoglobin, hematocrit, iron, and ferritin levels were higher in the IV iron group than the AWBT group. On POD 1, the levels of iron and ferritin were higher in the IV iron group than the AWBT group. On POD 2, the IV iron group showed lower hemoglobin level but higher levels of iron and ferritin compared to the AWBT group. Based on the values at 4 weeks before surgery, the levels of hemoglobin, hematocrit, and iron and RBC count decreased gradually during and early after surgery in both groups; however, the ferritin levels were continuously higher in the IV iron group than the AWBT group.

**Table 2:** Comparisons of perioperative red blood cell related findings between the two groups

Group	Intravenous iron	Autologous whole blood	*p*
n	32	32	
Four weeks before surgery (baseline)			
Hemoglobin (g/dL)	14.5 ± 1.4	14.3 ± 1.4	0.529
Hematocrit (%)	42.8 ± 3.8	42.5 ± 3.6	0.79
RBC count (× 10^12^/L)	4.8 ± 0.4	4.7 ± 0.4	0.491
Red cell index			
MCV (fL)	89.2 ± 3.5	89.3 ± 2.3	0.831
MCH (pg)	30.1 ± 1.3	30.1 ± 1.0	0.991
MCHC (%)	33.9 ± 0.9	33.5 ± 0.7	0.09
Iron (μg/dL)	128.9 ± 48.6	113.3 ± 42.4	0.176
Ferritin (ng/mL)	105.7 ± 92.9	107.5 ± 77.7	0.932
Immediately before surgery			
Hemoglobin (g/dL)	13.5 ± 1.4***	12.0 ± 2.0***	< 0.001
Hematocrit (%)	40.2 ± 2.7***	35.5 ± 5.5***	< 0.001
RBC count (× 10^12^/L)	4.5 ± 0.4***	4.0 ± 0.6***	< 0.001
Red cell index			
MCV (fL)	89.5 ± 3.4	89.1 ± 3.7	0.617
MCH (pg)	30.1 ± 1.3	30.0 ± 1.4	0.631
MCHC (%)	33.8 ± 0.8	33.6 ± 0.7	0.401
Iron (μg/dL)	98.6 ± 33.0***	62.2 ± 40.9***	< 0.001
Ferritin (ng/mL)	271.2 ± 156.4**	66.3 ± 111.6	< 0.001
Postoperative day 1			
Hemoglobin (g/dL)	10.1 ± 1.9***	10.1 ± 1.8***	0.849
Hematocrit (%)	30.1 ± 5.5***	29.7 ± 4.9***	0.765
RBC count (× 10^12^/L)	3.4 ± 0.6***	3.4 ± 0.6***	0.697
Red cell index			
MCV (fL)	89.0 ± 1.7	89.4 ± 3.3	0.542
MCH (pg)	30.0 ± 0.7	30.0 ± 1.2	0.829
MCHC (%)	33.7 ± 0.7	33.7 ± 0.8	0.961
Iron (μg/dL)	40.6 ± 29.8***	22.7 ± 12.1***	0.002
Ferritin (ng/mL)	265.2 ± 161.1***	102.3 ± 96.2	< 0.001
Postoperative day 2			
Hemoglobin (g/dL)	9.1 ± 1.6***	9.9 ± 1.4***	0.035
Hematocrit (%)	26.9 ± 4.9***	29.4 ± 4.3***	0.036
RBC count (× 10^12^/L)	3.0 ± 0.6***	3.3 ± 0.5***	0.022
Red cell index			
MCV (fL)	89.8 ± 2.5	89.9 ± 3.5	0.823
MCH (pg)	30.4 ± 1.0	29.9 ± 1.3	0.081
MCHC (%)	33.9 ± 0.5	33.7 ± 0.7	0.433
Iron (μg/dL)	63.0 ± 26.4***	41.4 ± 22.9***	0.001
Ferritin (ng/mL)	281.8 ± 150.3***	107.3 ± 93.5	< 0.001

Values are expressed as mean ± SD and number (proportion)

*RBC* red blood cell, *MCV* mean corpuscular volume, *MCH* mean corpuscular hemoglobin, *HCHC* mean corpuscular hemoglobin concentration

^*^p < 0.05 based on the level at 4 weeks before surgery (baseline)

^**^p < 0.01 based on the level at 4 weeks before surgery (baseline)

^***^p < 0.001 based on the level at 4 weeks before surgery (baseline)

**Fig. 2:**
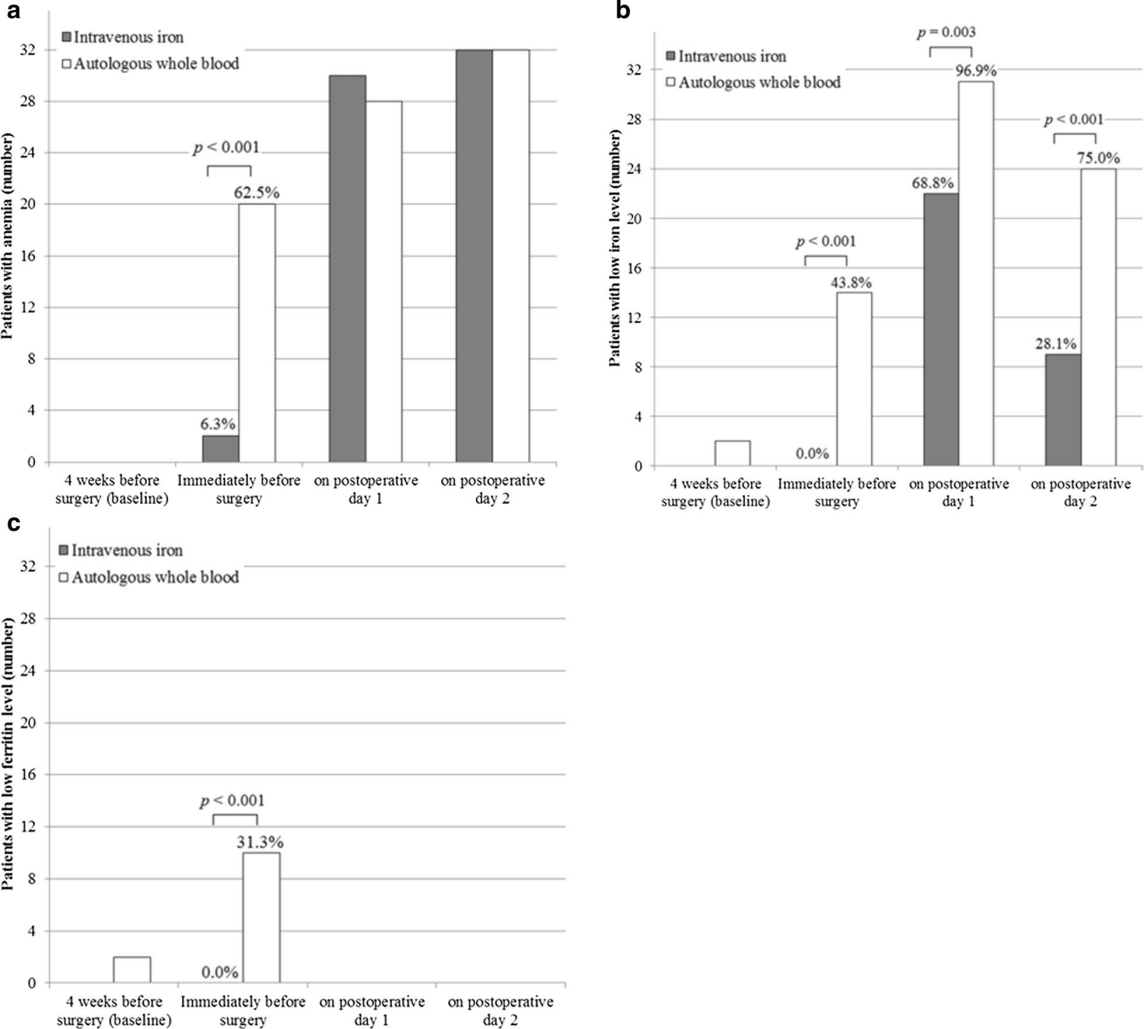
Comparison of proportion of patients with **a** anemia, and low levels of **b** iron and **c** ferritin at 4 weeks before surgery, immediately before surgery, and on postoperative days 1 and 2 in patients with IV iron therapy (*n* = 32) and autologous whole blood therapy (*n* = 32). Values are expressed as number and proportion (%)

The incidence of anemia immediately before surgery was lower in the IV iron group than the AWBT group (Fig. 2). The incidences of low iron level immediately before surgery and on PODs 1 and 2 were lower in the iron group than the AWBT group. The incidence of low ferritin level immediately before surgery was lower in the iron group than the AWBT group.

Based on the values at 4 weeks before surgery (baseline), changes in hemoglobin levels immediately before surgery were lower, but these changes on POD 2 were higher in the IV iron group than the AWBT group (Table 3). The change in iron level on POD 1 was lower in the iron group than the AWBT group. Changes in ferritin levels immediately before surgery and on PODs 1 and 2 were higher in the iron group than the AWBT group.

**Table 3:** Comparison of perioperative changes in hemoglobin, iron, and ferritin levels based on the values at 4 weeks before surgery (baseline) between the two groups

Group	Intravenous iron	Autologous whole blood	*p*
n	32	32	
Change of hemoglobin level (%)			
Immediately before surgery	− 6.47 ± 5.51	− 16.36 ± 10.27	< 0.001
Postoperative day 1	− 29.92 ± 11.21	− 29.73 ± 8.38	0.938
Postoperative day 2	− 37.18 ± 10.07	− 30.64 ± 7.02	0.004
Change of iron level (%)			
Immediately before surgery	− 14.78 ± 39.97	− 35.95 ± 54.82	0.082
Postoperative day 1	− 65.9 ± 23.04	− 76.26 ± 15.57	0.04
Postoperative day 2	− 43.57 ± 33.42	− 56.27 ± 35.9	0.148
Change of ferritin level (%)			
Immediately before surgery	273.28 ± 324.54	− 20.53 ± 213.09	< 0.001
Postoperative day 1	261.35 ± 281.57	45.38 ± 195.97	0.001
Postoperative day 2	309.07 ± 344.98	53.55 ± 188.3	0.001

Values are expressed as mean ± SD

Changes (%) are defined as ((each value—value at 4 weeks before surgery)/value at 4 weeks before surgery) × 100

### Noninferiority of differences in hemoglobin levels before, during, and after surgery between the two groups

**Fig. 3:**
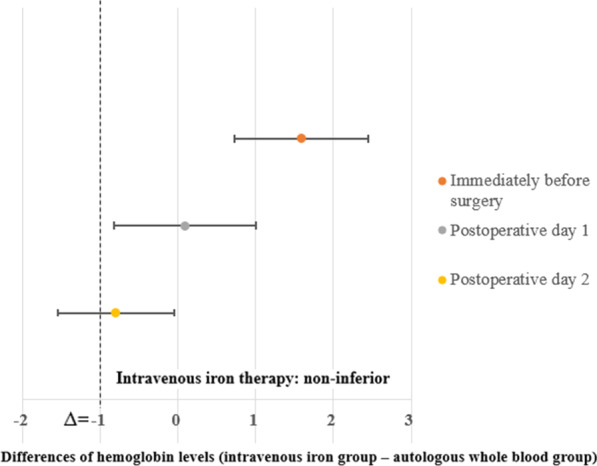
Noninferiority diagram of differences in hemoglobin levels between intravenous iron and autologous whole blood therapy groups immediately before surgery, and on postoperative days 1 and 2. A noninferiority margin (δ) indicates > Δ = − 1. Circles indicate mean hemoglobin differences and error bars indicate the 95% CIs of the differences between the two groups

The mean treatment difference (IV iron group—AWBT group) in the hemoglobin level on POD 1 between the two groups was 0.09 (95% CI = − 0.83 to 1.0) (Fig. 3). As the lower limit of the 95% CI (− 0.83) was higher than the prespecified noninferiority margin (δ = − 1), noninferiority was established. In addition, the mean treatment differences in hemoglobin levels between the two groups were as follows: immediately before surgery, 1.59 (95% CI = 0.73 to 2.44); and on POD 2, − 0.81 (95% CI = − 1.55 to − 0.06).

### Comparison of other postoperative findings between the two groups

The inflammatory findings before, during, and after surgery were comparable between the two groups (Additional file 3). With regard to coagulation findings, the platelet, antithrombin III, and fibrinogen levels on POD 1 were higher and the INR was more prolonged in the IV iron group than the AWBT group. On POD 2, the platelet and antithrombin III levels were lower and the INR were more prolonged in the IV iron group than the AWBT group. However, postoperative coagulation values were within the respective normal ranges in both groups [19, 26].

Although all patients experienced a mild degree (≤ 3 VAS) of discomfort or pain in both treatment groups, the median (IQR) VAS was lower in the IV iron group than the AWBT group (0 [0–0] vs. 2 [], respectively; *p* < 0.001). The proportion of patients with Clavien–Dindo classification II related to requirement of allogeneic PRBC transfusion on POD 2 was higher in the IV iron group than the AWBT group (*n* = 8, 25.0% vs. *n* = 1, 3.1%, respectively; *p* = 0.026). However, the total length of hospital stay was comparable between the two groups (2.5 ± 0.6 vs. 2.4 ± 0.5 days, respectively; *p* = 0.269).

### Discussion

We found that IV iron therapy provided noninferior hemoglobin levels compared to AWBT after bimaxillary orthognathic surgery in the first 24 h postoperatively. In the operating room, the patients with IV iron therapy showed higher levels of hemoglobin, iron, and ferritin than those with AWBT and they did not require allogeneic blood transfusion during surgery. In addition, the patients with IV iron infusion showed less discomfort/pain during the treatment compared to those from whom whole blood was harvested. However, the hemoglobin level in the IV iron group decreased markedly thereafter, and on the second day after surgery led to a greater need for blood transfusion.

Many observational studies have suggested benefits of early IV iron therapy that lead to regaining oxygen carrying capacity and improvement of organ functional recovery and quality of life in critically ill patients, such as those with kidney or heart failure [15, 16, 27]. Hemorrhage is one of the most common complications during surgery that causes acute loss of oxygen delivery capability and components, such as hemoglobin and iron. These losses are markedly aggravated when lost blood is replaced with fluid, and eventually have a negative impact on short- and long-term prognostic outcomes. In patients at risk for iron storage shortage or overt anemia, early IV iron therapy helps build up the levels of hemoglobin and iron preoperatively and reduce the need for blood transfusion intraoperatively [28, 29]. Piednoir et al. [10] reported that patients with iron deficiency but without anemia are associated with larger PRBC transfusions and poorer physical fatigue scores than those without iron deficiency or anemia after cardiac surgery. In Kim et al. [30], patients who received 500 mg or 1000 mg IV ferric carboxymaltose (ferric carboxymaltose group) had a better hemoglobin response, as determined by a hemoglobin increase ≥ 2 g/dL from baseline, and greater improvements in iron-related variables (i.e., serum levels of ferritin and levels of transferrin saturation) 12 weeks after gastrectomy than patients who received IV normal saline (placebo group). The requirement of alternative anemia care, including oral iron or transfusion, was lower in the ferric carboxymaltose group than in the placebo group. In addition, the occurrence and aggravation of anemia were affected by active and persistent inflammatory response related to conditions of absolute and/or functional iron deficiency after surgery. As these conditions of iron deficiency were hardly corrected by the administration of oral iron due to a decrease in gastrointestinal iron absorption related to surgical stimuli, IV iron administration seemed to be an efficient and safe means of enhancing recovery from anemia and attenuating the requirement for allogeneic blood transfusion with very few severe side effects, such as drug allergy [17]. In a study of anemic critically ill patients, Iperen et al. [31] suggested that the regime with IV iron sucrose at dose of 20 mg/day improved systemic inflammatory response with reduction in C-reactive protein level, and contributed to lowering both the requirement for blood transfusion and mortality rate.

However, commonly reported adverse events, associated with IV ferric carboxymaltose include injection site reactions, phlebitis, fever, nausea, constipation, headache, and diarrhea; reactions are mild to moderate in severity. In a large multicenter trial, the reported ferric carboxymaltose-related complications were injection site reactions (2.3%) and urticaria (2.3%), but there were no serious complications, such as hypersensitivity or anaphylactic reactions [30]. Among patients with chronic kidney dysfunction who received a higher dose of IV ferric carboxymaltose, two showed mild hypersensitivity that did not require hospitalization [32]. In women with a history of heavy uterine bleeding, an unexpected side-effect—hypophosphatemia—was reported, but no patients were symptomatic. Although this side-effect is not fully understood, it may be related to an increase in the phosphaturic hormone fibroblast growth factor-23 [33]. Although previous studies and our results demonstrate the safety of IV ferric carboxymaltose, there are still theoretical mechanisms by which IV iron could exacerbate cardiovascular injury due to its effect on oxidative stress, and potential concerns related to IV iron worsening infections and hypersensitivity [19, 32–34].

In contrast to previous studies, our findings did not show positive postoperative results of IV iron therapy, which may be explained by differences in patient condition and surgical settings. In many studies that have reported favorable outcomes of IV iron therapy, the patients were anemic or iron-deficient before surgery, with chronic and critical illnesses, such as malignancy, and older age. These factors disturbed iron balance and decreased oxygen delivery capability of hemoglobin. The correction of iron deficiency by IV iron infusion facilitated the recovery of iron-containing oxygen transport carriers, such as suitable structure and concentration of hemoglobin [10, 14–16, 28–30]. However, our patients were healthy, young, and did not have any comorbidities or abnormal laboratory variables preoperatively. Prophylactic IV iron treatment in these patients may not be as effective as in patients with existing iron-deficient anemia. In Oh et al. [35], the incidence of allogeneic blood transfusion was significantly lower in patients who received preoperatively collected autologous blood than in patients who did not. This difference was predominantly greater in patients with a preoperative hemoglobin level < 14 g/dL than ≥ 14 g/dL. However, the rate of discarding collected autologous blood was as high as 38.5% in patients with preoperative hemoglobin level ≥ 14 g/dL compared to the rate of 10.5% in those with a level < 14 g/dL (*p* < 0.0001). Therefore, appropriately raising the level of hemoglobin before surgery seems to be the cornerstone to successfully reducing the need for blood transfusion and the collection of large amounts of the patient’s own whole blood [30, 36, 37]. However, immediate recovery of hemoglobin level after surgery is not possible with IV iron administration, because production and maturation of hemoglobin derived from stored iron, such as ferritin, require several weeks to months [38]. Therefore, although patients who received AWBT had a higher incidence of anemia and lower levels of iron and ferritin before and during surgery than patients with IV iron therapy, the active response to regain hemoglobin level after intraoperative hemorrhage may be better in such patients.

The AWBT is a technique in which the patient’s own blood is collected and saved for a period of time before surgery, and reinfused during surgery. Repeated blood donations before surgery induce bone marrow cell proliferation and erythrocyte regeneration, which that accelerates hematopoietic function and postoperative recovery. These changes may decrease the requirement for allogeneic blood transfusion related to immunoreaction and infectious vulnerability [5, 39, 40]. However, the period from the first emergence of erythroid progenitor cells in the bone marrow to the emergence of mature erythrocytes in the peripheral blood is up to 4 weeks long, and erythrocyte regeneration in one withdrawn red blood cell unit takes up to 6 months [7–9]. Additionally, because of the limitations of conventional blood storage techniques using citrate–phosphate-dextrose-adenine solution for blood preservation and the red cell storage-life of 35 days at 4 °C, the AWBT should commence 4–5 weeks prior to surgery, allowing the collection of whole blood, and the last donation should take place at least 48–72 h before surgery to allow for equilibration of blood volume [21, 41, 42]. In patients who received the AWBT, the hematocrit and iron-related components significantly decreased from the initial level before AWBT to the level immediately before surgery [9]. Therefore, the AWBT is suitable primarily for young and robust patients without anemia scheduled for elective surgery in 4–5 weeks.

This study had some limitations. First, although IV iron infusion was performed 4 weeks before surgery, RBC synthesis may not function sufficiently during this period. Further studies are required to investigate the optimal interval between IV iron therapy and surgery in patients without overt anemia scheduled for surgery. Second, we were not able to determine the optimal dose of IV iron for prevention or compensation of the surgical loss in healthy patients, because there have been few studies on the relationship between type of surgery or bleeding and iron loss. Finally, we did not measure the long-term postoperative changes in hemoglobin, iron, and ferritin levels, such as those occurring over weeks to months. However, based on previously reported findings [30, 43], sufficient amounts of iron and ferritin in patients with IV iron therapy may play roles in restoring appropriate hemoglobin levels postoperatively. However, our study is the first to perform a non-inferiority comparison of the effect of a novel IV iron drug (ferric carboxymaltose) and conventional blood salvage therapy (autologous whole blood donation) on the postoperative hemoglobin level in robust young patients undergoing bimaxillary orthognathic surgery. Although previous studies suggested IV iron therapy ensured better oxygen-carrying capacity, those studies were limited by comparison to normal saline therapy as a control group.

## Conclusion

Although the levels of hemoglobin, iron, and ferritin were better immediately before surgery in healthy patients who received preoperative IV iron therapy, this treatment did not seem to be effective for significantly raising hemoglobin levels or reducing the low hemoglobin level-based requirement for allogeneic blood transfusion compared to AWBT after surgical hemorrhage and injury. However, as collection of autologous whole blood caused overt iron loss and anemia before surgery and intraoperative transfusion of the whole blood was unable to prevent the occurrence of persistent iron deficiency after surgery, IV iron therapy has potential benefits for iron homeostasis and subsequent erythropoiesis in healthy patients early after bimaxillary orthognathic surgery. Therefore, preoperative IV iron therapy alone may be equally effective in ensuring a sufficient hemoglobin level on POD 1 compared to therapy with intraoperative infusion of autologous whole blood harvested before surgery, but the non-inferiority results did not persist after POD 1. Appropriately powered randomized controlled trials examining the effects of IV iron with/without autologous whole blood and subsequent long-term outcome after bimaxillary orthognathic surgery are required.

## Supplementary Information

**Additional file 1.** Consolidated Standards of Reporting Trials (CONSORT) guidelines.

**Additional file 2.** Summary of our bimaxillary orthognathic surgery protocol.

**Additional file 3.** Comparison of perioperative inflammatory and coagulation findings between the two groups.

## Data Availability

The datasets used and/or analysed during this study are available from the corresponding author on reasonable request.

## Abbreviations

IV: Intravenous
POD: Postoperative day
ASA: American Society of Anesthesiologists
SBP: Systolic blood pressure
DBP: Diastolic blood pressure
HR: Heart rate
PRBC: Packed red blood cell
BMI: Body mass index
RBC: Red blood cell
INR: International normalized ratio
VAS: Visual analogue scale
IQR: Interquartile
