# Supplementary material for: Preemptive intravenous iron therapy versus autologous whole blood therapy for early postoperative hemoglobin level in patients undergoing bimaxillary orthognathic surgery: a prospective randomized noninferiority trial
Source: BMC Oral Health. 2021 Jan 7;21:16. doi: 10.1186/s12903-020-01359-1 (PMC7791750; doi:10.1186/s12903-020-01359-1)
Supplement: Supplementary file 2 — Additional file 2. Summary of our bimaxillary orthognathic surgery protocol. [file 12903_2020_1359_MOESM2_ESM.docx]

| **Additional file 2.** Summary of our bimaxillary orthognathic surgery protocol | | |
| --- | --- | --- |
| ***Preoperative day*** |  |  |
| Four weeks before surgery (baseline) | | |
| Inclusion and exclusion criteria | Inclusion criteria  1) adult (i.e., age ≥ 20 years)  2) elective bimaxillary orthognathic  surgery  3) ASA physical status I or II | Exclusion criteria  1) hemodynamic unstable case  2) ASA physical status III or IV  3) history of iron drug related side  effects (i.e., allergy)  4) medication history of anticoagulants  5) anemia  (i.e., hemoglobin level < 11 g/dL)  6) refusal to participate in the study |
| Randomization using sealed, opaque envelopes | | |
| Group | Intravenous iron group | Autologous whole blood group |
| Treatment | 1) 4 weeks before surgery  Single IV infusion of  ferric carboxymaltose (500 mg) | 1) 4 weeks before surgery  The 1^st^ collection of autologous whole blood (320 mL)  2) 2 weeks before surgery  The 2^nd^ collection of autologous whole blood (320 mL) |
| Measurement | 1) physical status  2) laboratory variables  3) visual analogue scale for discomfort/pain during each treatment | |
| ***Operation day*** |  |  |
| Immediately before surgery | | |
| Measurement | 1) laboratory variables | |
| During surgery |  | |
| Balanced anesthesia care | Induced hypotensive anesthesia  : adjustment of systolic blood pressure < 100 mmHg | |
| Replacement of hemorrhage | Colloid product | 2 units of autologous whole blood |
| Measurement | 1) hemodynamic variables  2) requirement of allogenic blood transfusion | |
| ***Postoperative day*** |  |  |
| On postoperative day 1 |  |  |
| Primary outcome | 1) hemoglobin level | |
| Secondary outcome | 1) laboratory variables  2) Clavien-Dindo classification  (i.e., requirement of allogenic blood transfusion) | |
| On postoperative day 2 | 1) laboratory variables  2) Clavien-Dindo classification  (i.e., requirement of allogenic blood transfusion)  3) total period of hospital administration | |
| **Abbreviation:** ASA, American Society of Anesthesiologists | | |
|  | | |
