# Supplementary material for: Preemptive intravenous iron therapy versus autologous whole blood therapy for early postoperative hemoglobin level in patients undergoing bimaxillary orthognathic surgery: a prospective randomized noninferiority trial
Source: BMC Oral Health. 2021 Jan 7;21:16. doi: 10.1186/s12903-020-01359-1 (PMC7791750; doi:10.1186/s12903-020-01359-1)
Supplement: Supplementary file 3 — Additional file 3. Comparison of perioperative inflammatory and coagulation findings between the two groups. [file 12903_2020_1359_MOESM3_ESM.docx]

| **Additional file 3.** Comparison of perioperative inflammatory and coagulation findings between the two groups | | | |
| --- | --- | --- | --- |
| **Group** | **Intravenous iron** | **Autologous whole blood** | ***p*** |
| **n** | **32** | **32** |  |
| ***Four weeks before surgery*** | | | |
| **Inflammatory findings** |  |  |  |
| C-reactive protein (mg/dL) | 0.19 ± 0.47 | 0.29 ± 0.92 | 0.579 |
| White blood cell count (x 10^9^/L) | 6.1 ± 0.9 | 5.9 ± 1.8 | 0.478 |
| Neutrophil (%) | 57.6 ± 6.7 | 55.4 ± 8.8 | 0.276 |
| Lymphocyte (%) | 33.1 ± 5.6 | 33.9 ± 8.3 | 0.629 |
| **Coagulation findings** |  |  |  |
| Platelet count (x 10^9^/L) | 263.6 ± 50.8 | 269.3 ± 66.5 | 0.702 |
| International normalized ratio | 1.04 ± 0.04 | 1.03 ± 0.04 | 0.47 |
| Activated partial thrombin time (sec) | 27.9 ± 2.2 | 28.4 ± 1.8 | 0.361 |
| Antithrombin III (%) | 102.3 ± 8.6 | 103.9 ± 6.8 | 0.409 |
| Fibrinogen (mg/dL) | 273.8 ± 72.8 | 275.1 ± 43.4 | 0.927 |
| ***Immediately before surgery*** | | | |
| **Inflammatory findings** |  |  |  |
| C-reactive protein (mg/dL) | 0.21 ± 0.37 | 0.1 ± 0.15 | 0.137 |
| White blood cell count (x 10^9^/L) | 5.8 ± 0.9 | 6.3 ± 2.6 | 0.273 |
| Neutrophil (%) | 58.2 ± 10.5 | 62.3 ± 12.1 | 0.158 |
| Lymphocyte (%) | 33.3 ± 8.5 | 29.1 ± 9.9 | 0.073 |
| **Coagulation findings** |  |  |  |
| Platelet count (x 10^9^/L) | 232.4 ± 38.4 | 240.2 ± 43.0 | 0.449 |
| International normalized ratio | 1.04 ± 0.07 | 1.05 ± 0.05 | 0.843 |
| Activated partial thrombin time (sec) | 28.6 ± 2.5 | 29.2 ± 3.0 | 0.291 |
| Antithrombin III (%) | 86.2 ± 7.2 | 89.1 ± 5.2 | 0.066 |
| Fibrinogen (mg/dL) | 231.4 ± 44.6 | 231.2 ± 54.0 | 0.986 |
| ***Postoperative day 1*** | | | |
| **Inflammatory findings** |  |  |  |
| C-reactive protein (mg/dL) | 4.42 ± 2.49 | 5.0 ± 1.44 | 0.258 |
| White blood cell count (x 10^9^/L) | 13.6 ± 3.2 | 12.6 ± 3.5 | 0.243 |
| Neutrophil (%) | 82.8 ± 5.1 | 83.6 ± 5.4 | 0.556 |
| Lymphocyte (%) | 9.1 ± 3.7 | 8.7 ± 4.5 | 0.696 |
| **Coagulation findings** |  |  |  |
| Platelet count (x 10^9^/L) | 186.8 ± 42.1 | 219.1 ± 65.6 | 0.023 |
| International normalized ratio | 1.14 ± 0.05 | 1.09 ± 0.07 | 0.006 |
| Activated partial thrombin time (sec) | 27.6 ± 3.0 | 27.7 ± 2.6 | 0.937 |
| Antithrombin III (%) | 87.6 ± 9.2 | 97.0 ± 10.0 | <0.001 |
| Fibrinogen (mg/dL) | 287.0 ± 53.0 | 341.3 ± 67.9 | 0.001 |
| ***Postoperative day 2*** | | | |
| **Inflammatory findings** |  |  |  |
| C-reactive protein (mg/dL) | 3.78 ± 2.39 | 3.19 ± 1.57 | 0.243 |
| White blood cell count (x 10^9^/L) | 11.1 ± 1.9 | 10.3 ± 2.5 | 0.147 |
| Neutrophil (%) | 78.8 ± 7.7 | 74.6 ± 10.9 | 0.08 |
| Lymphocyte (%) | 12.9 ± 7.2 | 17.0 ± 9.9 | 0.063 |
| **Coagulation findings** |  |  |  |
| Platelet count (x 10^9^/L) | 170.4 ± 47.2 | 209.5 ± 51.6 | 0.002 |
| International normalized ratio | 1.09 ± 0.05 | 1.06 ± 0.04 | 0.001 |
| Activated partial thrombin time (sec) | 26.7 ± 2.4 | 27.1 ± 2.2 | 0.486 |
| Antithrombin III (%) | 91.7 ± 8.2 | 101.4 ± 7.9 | <0.001 |
| Fibrinogen (mg/dL) | 350.4 ± 32.6 | 367.6 ± 52.9 | 0.124 |
| **NOTE:** Values are expressed as mean ±SD and number (proportion). | | | |
